# Dynamic Bandwidth Part Allocation in 5G Ultra Reliable Low Latency Communication for Unmanned Aerial Vehicles with High Data Rate Traffic

**DOI:** 10.3390/s21041308

**Published:** 2021-02-12

**Authors:** Minsig Han, Jaewon Lee, Minjoong Rim, Chung G. Kang

**Affiliations:** 1 School of Electrical Engineering, Korea University, Seoul 02841, Korea; als4585@korea.ac.kr (M.H.); lijrew@korea.ac.kr (J.L.); 2 Department of Information and Communication Engineering, Dongguk University, Seoul 02841, Korea; minjoong@dongguk.edu

**Keywords:** URLLC, eMBB, unmanned aerial vehicle, bandwidth part, resource allocation, dynamic multiplexing, orthogonal slicing

## Abstract

The 3GPP standardized the physical layer specification in 5G New Radio to support enhanced mobile broadband (eMBB) and ultra-reliable low-latency communication (URLLC) coexistence in usage scenarios including aerial vehicles (AVs). Dynamic multiplexing of URLLC traffic was standardized to increase the outage capacity. DM allocates a fully overlapped bandwidth part (BWP) of eMBB and URLLC AVs to perform the immediate scheduling of URLLC traffic by puncturing ongoing eMBB traffic. However, DM often suffers from a significant frame error incurred by puncturing. Meanwhile, BWP can be sliced orthogonally for eMBB and URLLC AVs, possibly preventing overdimensioning the resources depending on the eMBB and URLLC traffic loads. In this paper, we propose a dynamic BWP allocation scheme that switches between two multiplexing methods, dynamic multiplexing (DM) and orthogonal slicing (OS), so as to minimize an impact of uRLLC traffic on eMBB traffic. To implement efficient BWP allocation, the capacity region is analyzed by considering the effect of physical layer parameters, such as modulation and coding scheme (MCS) levels and code block group size on DM and OS. OS is effective for improving the eMBB throughput under a URLLC latency constraint for deterministic and predictable URLLC traffic, whereas DM has limited error-correcting capability against the URLLC’s puncturing effect. The relative MCS level of eMBB and URLLC is critical in determining the eMBB traffic tolerance against puncturing. Identifying the performance tradeoff between DM and OS, the tolerance level is quantified by a URLLC load threshold. It is given in an approximate closed form, which is an essential reference for selecting DM over OS, enabling dynamic BWP allocation for the URLLC AV.

## 1. Introduction

The effective coexistence of high data rate traffic and extremely reliable traffic with low latency is a vital feature for the successful commercialization of aerial vehicles (AVs) [1]. For example, in an unmanned aerial vehicle (UAV) system, numerous drones should be able to transmit real-time high-definition video traffic to the surrounding base stations, while their operations are controlled by highly reliable downlink traffic with low latency. In contrast, a personal aerial vehicle, which is also known as a flying car, requires an aircraft to transmit and receive highly reliable autonomous flight messages with low latency for command and control while receiving video streaming services over downlink broadband traffic. Such a coexistence of diverse traffic causes significant changes in the physical layer design compared to the previous generation of communication systems.

In the 3GPP New Radio (NR) specification for 5G, two different usage scenarios, namely enhanced mobile broadband (eMBB) and ultra-reliable low-latency communication (URLLC) services, are supported for high data rate traffic and low-latency/high-reliability traffic, respectively. Each service scenario has its own quality of service (QoS) requirements that need to be satisfied. More specifically, the data rate for eMBB services must be maximized while satisfying two conflicting requirements for URLLC service (i.e., latency and reliability). The NR system aims to efficiently support diverse AVs with different usage scenarios in a single carrier bandwidth (CBW). To this end, it includes new physical layer specifications, such as numerologies for subcarrier spacings (SCS) and slot length, code block group (CBG)-based transmission, bandwidth part (BWP), and preemption-based URLLC scheduling. Along with other physical layer specifications, a preemption-based scheduling mechanism allows URLLC traffic to be immediately scheduled by puncturing or superposing ongoing eMBB traffic. Notably, the preemption-based scheduling in 3GPP is also referred to as eMBB/URLLC dynamic multiplexing (DM) in the literature. A detailed description of the eMBB/URLLC DM (or simply DM) was reported previously [2].

The concept of DM was first introduced in [3], wherein it was argued that URLLC traffic with Poisson arrival should be wideband allocated to increase capacity while satisfying the outage capacity of URLLC. Furthermore, because of the sporadic nature of URLLC, traffic tends to underutilize a given wideband resource and it would be more efficient to schedule eMBB traffic in resources that are not occupied by URLLC traffic. That is, the motivation of DM in [3] was to maximize the outage capacity of URLLC traffic while increasing the resource utilization of the system. However, in [3], only the URLLC outage capacity and system resource utilization were considered in DM without considering the throughput of eMBB traffic or eMBB/URLLC sum throughput. More efficient implementation of eMBB/URLLC DM has been considered in various studies [4,5,6,7]. For example, Ref. [4] proposed recovery mechanisms for punctured eMBB traffic as well as a service-specific scheduling policy and link adaptation for the efficient implementation of eMBB/ URLLC DM. Meanwhile, Ref. [5] presented three eMBB traffic loss models by puncturing or superposing URLLC traffic while providing an individual optimal URLLC scheduler for each loss model. The optimal schedulers simultaneously maximize the utility of the eMBB traffic subject to the URLLC demand requirement. In [6], the latency of URLLC traffic was reduced from 1.3 to 1 ms, without incurring an eMBB performance loss of more than 10%, by the joint link adaptation of eMBB and URLLC traffic, which adjusts the block error probability of the URLLC payload. In [7], preemption-aware rank offloading scheduling was proposed to increase the overall ergodic capacity while reducing the latency of URLLC traffic with enhanced scheduling flexibility.

A common objective of eMBB/URLLC DM in previous studies [2,3,4,5,6,7] is to minimize eMBB performance loss while satisfying the latency and reliability requirements of URLLC traffic. However, this is not essential for either the throughput maximization of eMBB or the delay guarantee of URLLC. Depending on the conditions, the same goal can be achieved by using eMBB/URLLC orthogonal slicing (OS), which divides the resources exclusively for each type of traffic according to their traffic load. There are some obvious cases in which the OS is more appropriate than eMBB/URLLC DM. For example, when a URLLC AV has a fixed and predictable traffic load in a given period, then an exact amount of resource is orthogonally sliced for the specific URLLC AV without incurring a penalty for URLLC traffic. Meanwhile, eMBB traffic errors due to the puncturing of URLLC traffic must be detected and corrected through channel coding/decoding. Because channel coding can only fix a limited number of erased bits, DM incurs a significant throughput loss for eMBB as long as the multiplexed URLLC traffic exceeds a certain limit.

As previously discussed, there are two multiplexing methods to achieve the coexistence of eMBB and URLLC, namely DM and OS; the preference for a method depends on the situation. We first reveal the underlying performance characteristics of these options, which must be identified to allow for dynamic switching between them. As discussed in Section 3, switching between DM and OS involves with BWP allocation to determine which BWP is used for URLLC AVs. Furthermore, the physical layer parameters are comprehensively explored to observe the performance tradeoff between DM and OS. To implement dynamic switching of DM and OS, the base station must know in advance which multiplexing methods outperforms the other. In this paper, we propose a dynamic BWP allocation scheme that switches between two multiplexing methods, dynamic multiplexing (DM) and orthogonal slicing (OS), so as to minimize the impact of URLLC traffic on eMBB traffic. To this end, we present a closed form expression of the URLLC load as an optimal tradeoff threshold at which their performance is reversed. Using this threshold, DM and OS can be switched to minimize an impact of URLLC traffic on eMBB traffic. While BWP is a key physical layer feature of to support flexibility and power saving in higher spectrum [8], there has been no embodiment of dynamic BWP allocation schemes that achieves near-optimal performance tradeoff by exploiting an explicit threshold that varies with the physical parameters. Our key contribution in this paper is to identify its performance tradeoff with analytical model of the capacity region, which allows for deriving an approximate closed-form expression of switching threshold for URLLC traffic load.

The remainder of this paper is organized as follows. Section 2 reviews 3GPP specifications for the efficient coexistence of eMBB and URLLC. Section 3 presents the system models for DM and OS, which forms the basis for the throughput analysis of eMBB presented in Section 4. Section 5 presents the numerical results for DM and OS subject to the various physical layer parameters. Finally, the conclusions are presented in the last section.

## 2. Preliminaries on the 3GPP NR Physical Layer Specification

In the NR specification for 5G, AVs with eMBB and URLLC traffic should coexist efficiently in a single carrier bandwidth (CBW). Accordingly, the standardization activities of 3GPP aim to support these AVs with different requirements. Among the various improvements that can be made to achieve efficient coexistence, we focused on the physical layer enhancements standardized in 3GPP until the recent Release 16 specification in this study.

### 2.1. Use Cases and Their Requirements

Reliability and user-plane latency are the new physical layer requirements in URLLC systems. The reliability requirements are defined to ensure that a 32-byte URLLC packet is successfully transmitted with a success probability of $1-10^{-5}\left(99.999\%\right)$ with a user-plane latency of 1 ms [9]. The user-plane latency includes scheduling delay, queuing delay, transmission delay, receiver-side processing, and decoding delay, as well as multiple round-trip times for hybrid automatic repeat request (HARQ).

Before supporting a variety of URLLC use cases in the future releases, prior use cases such as AR/VR, factory automation, transport industry, and power distribution were specified in Release 16 [10]. Moreover, command and control communication for UAVs is another important use case. As individual use cases have their own requirements, physical layer enhancement has been specified to improve URLLC performance. Table 1 illustrates the generally considered requirements for the evaluation of individual use cases, including one for command and control in the UAV network. The following subsection shows the physical layer aspects specified for supporting URLLC traffic while allowing for efficient means of coexistence between URLLC and eMBB traffic in various use cases.

### 2.2. Numerologies for eMBB and URLLC Traffic

To satisfy the high reliability requirements of URLLC traffic, it is necessary to have multiple HARQ retransmissions or PDSCH repetition, which may be impossible to transmit as much as desired within a transmission time interval (TTI) of 1 ms. A TTI of 1 ms is equivalent to a subcarrier spacing (SCS) of 15 kHz, which is a default slot structure in LTE. Therefore, the 1 ms TTI should be shortened to enable multiple transmissions across multiple TTIs to meet the reliability requirements within 1 ms. In 5G NR numerology, the TTI is determined in terms of the parameter μ∈[0,⋯,4], which allows for scalable subcarrier spacing. More specifically, TTI can be shortened to ${\left(1/2\right)}^{\mu }\cdot 1\;\mathrm{ms}$ while SCS is expanded as $2^{\mu }\cdot 15\;\mathrm{kHz}$. In fact, the TTI for eMBB traffic remains 1 ms, i.e., μ=0, while that for URLLC traffic can be shortened, μ=1,2,3,4. Therefore, in the same CBW, two slot structures of different numerologies are allowed for the coexistence of eMBB and URLLC traffic.

### 2.3. Bandwidth Part

A notion of BWP is introduced in 5G NR to support the variable channel bandwidth and SCS of different AVs. Each BWP consists of one or more consecutive sets of continuous physical resource blocks within the CBW with its own numerology. Each AV can be configured with a maximum of 4 BWP for downlink and uplink; however, at a given point of time, only one BWP is active for downlink and one for uplink. The AVs are informed about the active BWP using a radio resource control message. AVs are not expected to transmit or receive control messages or data traffic outside of their active BWP. The BWP concept enables AVs to operate in a narrow or wide bandwidth depending on their demand, with several numerologies associated with their BWP. Because different BWPs overlap in the same CBW, BWPs with several different numerologies can coexist in one CBW [11].

### 2.4. Preemption-Based Dynamic Multiplexing of URLLC and eMBB Traffic

When the URLLC traffic arrives sporadically in the AV, all resources may not be fully utilized in the given BWP. Therefore, the DM of eMBB and URLLC traffic in AVs is expected to enhance resource utilization [2]. Furthermore, the URLLC traffic should be scheduled immediately within the delay limit. For this, it is allowed to schedule URLLC traffic over resources that have already been scheduled for eMBB traffic. There are two ways to achieve DM in the 3GPP specification: transmission of URLLC traffic by preemption (puncturing) of ongoing eMBB traffic and superposition of URLLC traffic over eMBB traffic. For the second option, an advanced receiver is required to detect superposed signals.

In this study, we assumed preemption-based scheduling as the baseline DM scheme. Even though URLLC traffic would benefit from its excellent resource utilization and outage capacity, the performance of eMBB traffic can be severely degraded [12]. To alleviate this performance degradation, a CBG-based transmission and retransmission scheme is introduced in the 3GPP specification, which is detailed in the following subsection. Furthermore, the control information of the preemption indicator is given in 14-bit bitmap, and 1-bit code block group flush out information is specified to indicate the preempted resource while flushing out the corrupted resources by URLLC preemption [13]. This is utilized to prevent the eMBB AV from decoding preempted resources.

### 2.5. Code Block Group-Based Transmission

CBG-based transmission is another important feature required for the implementation of preemption-based DM. In general, an entire transport block (TB) must be re-transmitted if a single code block (CB) is in error. Therefore, resource utilization can be seriously degraded when the TB size increases. Instead, the retransmission unit can be reduced, for example, in a unit of CB, to handle the inefficiency of retransmissions in a unit of TB. However, CB-by-CB retransmission may suffer from a serious control overhead of ACK/NACK reporting for individual CBs. To reduce such an overhead, 3GPP defines a CBG, which is a group of multiple CBs, as a basic retransmission unit.

**Figure 1:**
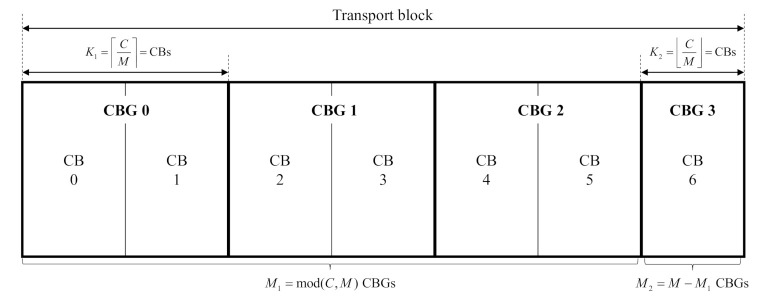
Grouping of CBG: Example for C=7 and N=4.

Figure 1 illustrates how each TB is constructed by grouping multiple CBGs, i.e., *M* CBGs with each CBG of C CBs. When the maximum number of CBGs that one TB can contain is *N*, the number of CBGs in the TB is determined as M=min(N,C). The *M* CBGs are divided as follows: $M_{1}$ CBGs having the same size and the remining $M_{2}$ CBGs. That is, $M_{1}=\;\mathrm{mod}\;\left(C,M\right)$ and $M_{2}=M-M_{1}$. The number of CBs in $M_{1}$ is given as $K_{1}=\left\lceil C/M\right\rceil$, and the CBs in $M_{2}$ is $K_{2}=\left\lfloor C/M\right\rfloor$. When the transmission fails, the gNB does not retransmit an entire TB consisting of M CBGs; instead, it transmits only the failed CBGs. For this reason, gNB carries 0, 2, 4, 6, or 8 bits of code block group transmission information (CBGTI), which has an in-order one-to-one mapping with M CBGs of the TB, with the MSB mapped to the first CBG [14]. A value of 0 or 1 in the CBGTI field indicates that the corresponding CBG is not transmitted or is to be transmitted, respectively.

## 3. System Model for Dynamic Multiplexing and Orthogonal Slicing

Let $U_{eMBB}$ and $U_{URLLC}$ denote a set of eMBB and URLLC AVs, respectively. In this study, we considered a downlink system in which the AVs in $U_{eMBB}$ and $U_{URLLC}$ share a single CBW. Let Φ and $\phi_{u}$ denote the number of RBs in the CBW and the number of RBs allocated to the AV *u*, respectively. The common BWP size for eMBB AVs is the same as that for CBW, i.e., $\Phi =\sum_{u\in U_{eMBB}}\phi_{u}^{}$. We assumed a full buffer model for eMBB AVs, implying that eMBB AVs always have data to transmit. Further, we assumed a non-full buffer model for URLLC AVs, in which packets of a fixed size *P* (bits) arrive with a Poisson distribution with an average rate of λ (the number of packets/ms/user). The average arrival rate λ was assumed to change only at each eMBB slot boundary and can be known in advance. Then, the traffic load of the URLLC load, which is denoted as *D*, can be defined as the total number of bits arriving during each eMBB slot. For a given TTI parameter μ, it is given as

(1) $$D=\left|U_{URLLC}\right|\cdot \lambda \cdot P\cdot {\left(1/2\right)}^{\mu }$$

One eMBB slot is divided into $L=2^{\mu }$ URLLC slots. The eMBB AV transmits one TB with $N_{CBG}$ CBGs in each eMBB slot of $\phi_{u}$ RBs. $N_{CBG}$ CBGs are encoded using the maximum distance separable (MDS) code with a code rate r=k/n. Low-density parity check (LDPC) code can be constructed based on the MDS code [15].

**Figure 2:**
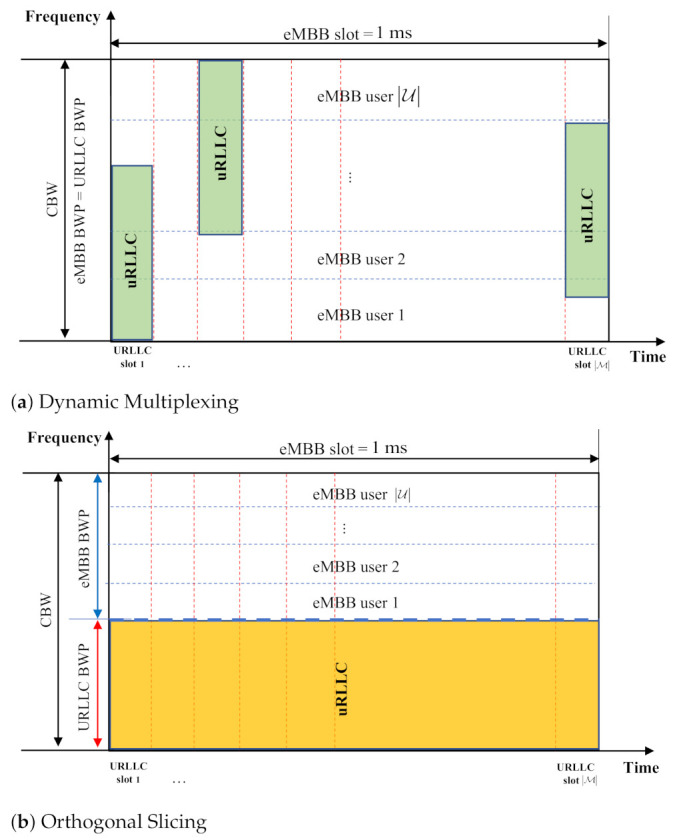
Two different eMBB/URLLC multiplexing: DM vs. OS.

Figure 2 illustrates how URLLC traffic is multiplexed over the ongoing eMBB traffic for DM and OS-based coexistence within an eMBB slot. The eMBB AV *u* is scheduled in units of eMBB slots in the time domain and $\phi_{u}$ consecutive RBs in the frequency domain. First, in DM-based URLLC multiplexing, the URLLC traffic should be scheduled as soon as the traffic arrives. In addition, we assumed that URLLC AVs have a BWP size equal to the CBW, which minimizes the effect of puncturing of eMBB traffic. In a given BWP, URLLC traffic is assumed to be scheduled in a uniform distribution over its allocated BWP preempting the eMBB resource as shown in Figure 2a. As URLLC AVs cannot be informed about the ongoing eMBB traffic, the uniform preemption of URLLC traffic is most acceptable for immediate scheduling.

In OS-based URLLC multiplexing, the resources for eMBB and URLLC AVs are orthogonally sliced, as shown in Figure 2b. Therefore, URLLC AVs are allocated with an exact size of BWP to support a load of *D* with a part of the eMBB slot, while the eMBB BWP occupies the remaining resources within the CBW. It is expected that the larger the URLLC load, the lower the eMBB throughput performance in both DM and OS, with an obvious performance tradeoff between them. In the subsequent section, the throughput performance of eMBB traffic subject to URLLC multiplexing is analyzed in terms of the various parameters involved in DM and OS, while discussing the size of URLLC BWP.

## 4. Dynamic BWP Allocation: Dynamic Multiplexing vs. Orthogonal Slicing

### 4.1. Throuhput Analysis for Dynamic Multiplexing and Orthogonal Slicing

In DM-based URLLC multiplexing, the eMBB AV flushes out the corresponding resources where the URLLC overlaps. Owing to the effect of flushing, the bits of the corresponding eMBB CBG are partially erased. To compensate for the erased bits, we assumed that eMBB traffic is encoded with an MDS code at a coding rate of r=k/n, which has the error-correction ability to fix *l* erased errors. Because the MDS code achieves the Singleton bound, it has a minimum distance of d=n−k+1, which can correct l=d−1=n−k erased bits [16]. Furthermore, it is assumed that ideal scrambling is applied to the RBs assigned to the CBGs that belong to one TB of eMBB AVs; therefore, the erased bits in each CBG are uniformly distributed, and the locations of the erased bits can be known.

**Figure 3:**
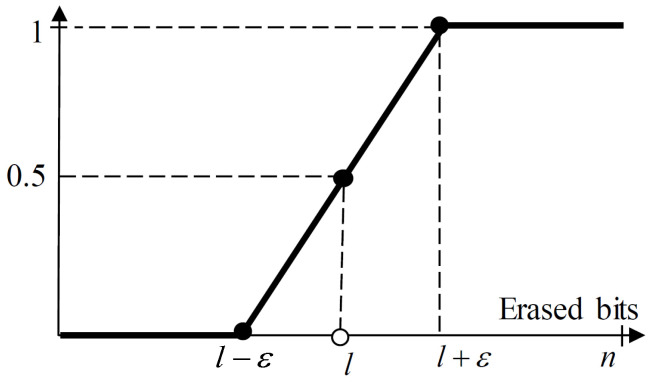
Modeling of error rate for code block group (CBG).

Because the MDS code for CBG can fix *l* erased bits, we can model the CBG error rate (CBGER) function with a shifted unit step function. In NR specification, CBG is constructed by low density parity check (LDPC) code for shared channel. As LDPC code is a kind of MDS code; (n,k) code can correct n−k bits [16]. If the number of bits in error incurred by puncturing is less than n−k bits, therefore, the CBG error rate is 0. Otherwise, the CBG error rate is 1. Therefore, the CBG error rate can be modeled as a unit step function. In order to obtain our switching threshold, $D_{threshold}$, for dynamic BWP allocation scheme, however, we expect the CBG error rate to be a continuous function. To maintain continuity in the CBGER function, we approximate it as follows:

(2) $$P_{CBGER}\left(x\right)=\left\{\begin{array}{c} \begin{array}{c} 0,\;x<l-\varepsilon \\ \frac{1}{2\varepsilon }\left(x-l\right)+\frac{1}{2},\;l-\varepsilon \leq x\leq l+\varepsilon \end{array}\\ 1,\;l+\varepsilon <x \end{array}\right.$$

where *x* denotes a number of erased bits in CBG and ε is zero or positive integer value indicating a margin for the bit-error-correction capability *l*. Figure 3 illustrates CBGER, which approaches a shifted unit step function as ε tends to zero. Once $D_{threshold}$ is computed with the CBRER function with a parameter ε in Figure 3, an approximate closed-form expression for near-optimal threshold, $D_{threshold}$, for LDPC code can be obtained by setting ε to zero, making the CBRER function close to a unit step function, as required for LDPC code.

Furthermore, if we assume that all eMBB AVs are assigned the same number of RBs, the number of bits erased by URLLC traffic in each eMBB CBG is given as

(3) $$\delta_{CBG}=\frac{\phi_{u}\cdot b\left(I_{MCS}^{\left(u\right)}\right)}{N_{CBG}}\cdot \frac{\Phi_{URLLC}}{\Phi }=\frac{\phi_{u}\cdot b\left(I_{MCS}^{\left(u\right)}\right)}{N_{CBG}}\cdot \frac{D}{\Phi \cdot b(I_{MCS}^{URLLC})}$$

where $b(I_{MCS}^{\left(u\right)})$ and $b(I_{MCS}^{URLLC})$ are the number of bits per RB when the modulation and coding scheme (MCS) index for AV *u* is given by $I_{MCS}^{\left(u\right)}$ and the MCS index for all the URLLC AVs is given as $I_{MCS}^{URLLC}$. Considering (2), the throughput of an eMBB AV is given as

(4) $$\sum_{u\in U_{eMBB}}\phi_{u}\cdot b\left(I_{MCS}^{\left(u\right)}\right)\cdot \left(1-P_{CBGER}\left(\delta_{CBG}\right)\right)$$

From (4), the throughput of one CBG in an eMBB AV *u* in the DM is given as

(5) $$\frac{\phi_{u}b\left(I_{MCS}^{\left(u\right)}\right)}{N_{CBG}}\cdot \left(1-P_{CBGER}\left(\delta_{CBG}\right)\right)$$

In OS-based URLLC multiplexing, on the other hand, the resource is orthogonally sliced in proportion to the URLLC load *D*. If all URLLC AVs have an MCS of $I_{MCS}^{URLLC}$, the number of RBs assigned to URLLC $\Phi_{URLLC}$ is given by $\Phi_{URLLC}=D/b\left(I_{MCS}^{URLLC}\right)$. Consequently, the number of RBs assigned to eMBB AVs is $\Phi_{eMBB}=\Phi -\Phi_{URLLC}$, and the number of RBs assigned to eMBB AV decreases at a rate of $\left(\Phi -\Phi_{URLLC}\right)/\Phi$. Then, the number of bits for the eMBB AV u is given as

(6) $$r_{u}=\phi_{u}\cdot b\left(I_{MCS,u}\right)\cdot \left(\Phi -\Phi_{URLLC}\right)/\Phi$$

Then, the throughput of the entire eMBB AV is given as follows:

(7) $$\sum_{u\in U_{eMBB}}\phi_{u}\cdot b\left(I_{MCS,u}\right)\cdot \left(\Phi -\Phi_{URLLC}\right)/\Phi$$

Furthermore, the throughput of one CBG in an eMBB AV *u* in OS is given as

(8) $$\frac{\phi_{u}\cdot b\left(I_{MCS}^{\left(u\right)}\right)}{N_{CBG}}\cdot \left(1-\frac{D}{b(I_{MCS}^{URLLC})}\cdot \frac{1}{\Phi }\right)$$

Comparing (5) with (8), when the URLLC load increases, the performance degradation of eMBB becomes more severe in DM than in OS. This is attributed to the fact that DM relies on the error-correction capability of the CBG-based MDS code to compensate for the performance loss. However, when the URLLC load is small, the punctured bit can be properly corrected using MDS code. Therefore, we expect that a critical level of URLLC load exists, such that OS outperforms DM.

We denote the threshold of URLLC load as $D_{threshold}$, where the eMBB throughput of OS outperforms that of DM. To determine $D_{threshold}$, we can analytically obtain the value that achieves equality in the following inequality:

(9) $$1-P_{CBGER}\left(\delta_{CBG}\right)\leq 1-\frac{D}{b(I_{MCS}^{URLLC})}\cdot \frac{1}{\Phi }$$

where only the comparison between the latter part of the eMBB throughput given in (5) and (8) is shown. Furthermore, by calculating (9), the following theorem is established:

**Theorem** **1**. *In the given system model, $D_{threshold}$ can be expressed as follows:*

(10) $$D_{threshold}=N_{CBG}\cdot l\cdot \left(\frac{\Phi }{\phi_{u}}\right)\cdot \left(\frac{b(I_{MCS}^{URLLC})}{b(I_{MCS}^{\left(u\right)})}\right)$$

**Proof** **of** **Theorem** **1**. See Appendix A. □

Equation (10) affords a closed-form value of the threshold of one CBG for an eMBB AV, given in our NR-based system model. Therefore, the proportional relationships and average tendency of $D_{threshold}$ can be exploited to switchingreferenced to determine between DM and OS. As shown in (10), the value of $D_{threshold}$ tends to increase with an increase in the number of CBGs $N_{CBG}$ and the relative MCS of eMBB and URLLC $b\left(I_{MCS}^{URLLC}\right)/b\left(I_{MCS}^{\left(u\right)}\right)$. In the following subsection, the performance of eMBB throughput for DM and OS is analyzed by varying the URLLC load to verify the relationship shown in (10).

### 4.2. Simulation Results

In this section, the throughput of eMBB was calculated using Monte Carlo simulations in a downlink system where eMBB and URLLC AVs coexist to verify the proportional relationship given in (10). We assumed ten eMBB AVs and one URLLC AV, which share a CBW of 100 RBs. In dynamic multiplexing, impact of URLLC to eMBB is mainly governed by how much eMBB resources are punctured by URLLC traffic. The amount of eMBB resources that are punctured does not depend on OFDM numerology of URLLC. It is attributed to the fact that the amount of time-domain resources reduced by the larger SCS is equivalent to that of frequency-domain resources increased [17]. As we are assuming an ideal interleaving for eMBB, its performance is not variant as long as the same amount of resources are maintained. It implies that our performance would be the same for the different numerologies with the different SCS, i.e., requiring the same D threshold. Without loss of generality, therefore, we just assume the same OFDM numerologies with μ=0 for both eMBB and URLLC traffic in the current analysis. To focus only on the throughput performance, control overheads such as reference signal, preemption indication, and HARQ retransmissions were not considered explicitly in the current analysis. In particular, however, we can still assume that retransmission load is a part of a traffic load considered in the simulation [18,19,20].

The URLLC traffic would be mostly originated by time-critical applications, e.g., flight control messages, with random arrivals [21]. More specifically in our simulation, we assume a full buffer model for eMBB traffic with a fixed high data rate while employing a Poisson arrival model for URLLC traffic. Furthermore, we adopt lower levels of MCS for URLLC service, which usually requires a robust performance. Table 2 presents the MCS index table for the physical downlink shared channel (PSDCH) of both eMBB and URLLC AVs as prescribed in the 3GPP specification [22]. In our simulation, the MCS index of URLLC AV, which is denoted as $I_{MCS}^{URLLC}$, is set to have one of the low MCS levels with QPSK modulation, i.e., $I_{MCS}^{URLLC}=0,1,2,\ldots ,9$, for the most robust performance.

**Figure 4:**
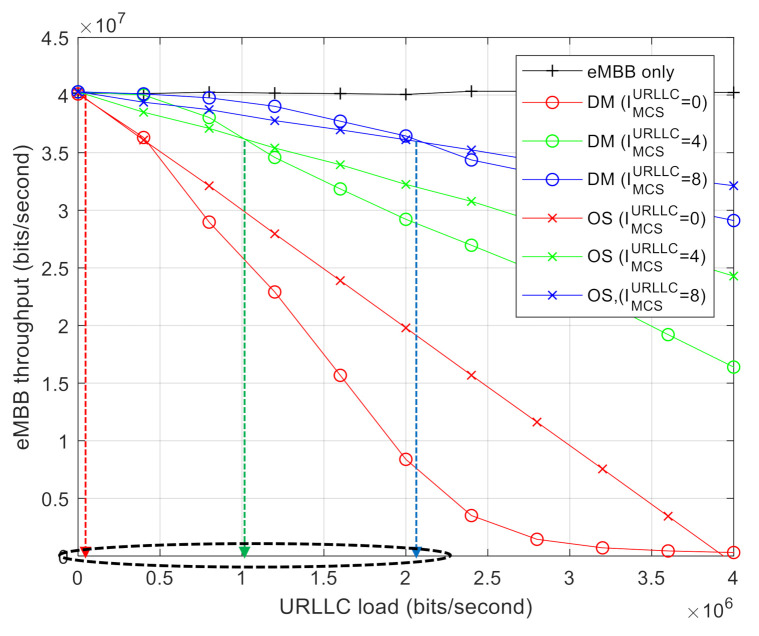
Capacity region with varying the URLLC MCS index: DM vs. OS.

Figure 4 shows the capacity regions of DM and OS. It indicates the throughput of eMBB AVs obtained by changing the traffic load *D*. The capacity regions were observed by varying the URLLC MCS index and assigning a random MCS index for eMBB, which is uniformly distributed over all possible MCS indices from 0 to 28. The code rate of MDS code for the given MCS index of eMBB also follows Table 2. Meanwhile, we considered $I_{MCS}^{URLLC}$ = 0, 4, and 8. It is obvious that the throughput of eMBB improves as $I_{MCS}^{URLLC}$ increases, thereby requiring less resources for URLLC AV. As shown in Figure 4, a higher MCS index increases the capacity region, where DM outperforms OS. Furthermore, DM outperforms OS up to a certain level of URLLC load, which implies that OS becomes more effective when a system is overloaded with URLLC traffic. In other words, there exists a threshold level of URLLC load, denoted as $D_{threshold}$, which can be a measure of durability of eMBB traffic against puncturing by URLLC traffic. Because the URLLC traffic requires less resources to achieve a higher MCS level, $D_{threshold}$ increases with higher MCS indices of URLLC AV.

**Figure 5:**
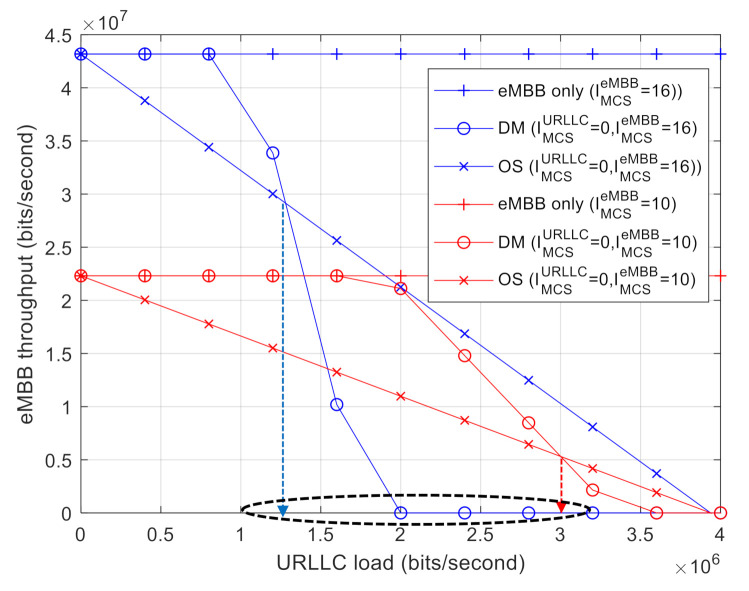
Capacity region with varying the eMBB MCS index: DM vs. OS.

Figure 5 shows the capacity regions for DM and OS while varying the MCS level of eMBB AVs. More specifically, we set $I_{MCS}^{eMBB}=10$ and $I_{MCS}^{eMBB}=16$ while fixing $I_{MCS}^{URLLC}=0$. It was found that $D_{threshold}$ increases with smaller eMBB MCS indices as DM becomes more tolerable against puncturing because of the increased error-correcting capability. From the results shown in Figure 4 and Figure 5, it is clear that $D_{threshold}$ is governed by a relative MCS level between eMBB and URLLC.

**Figure 6:**
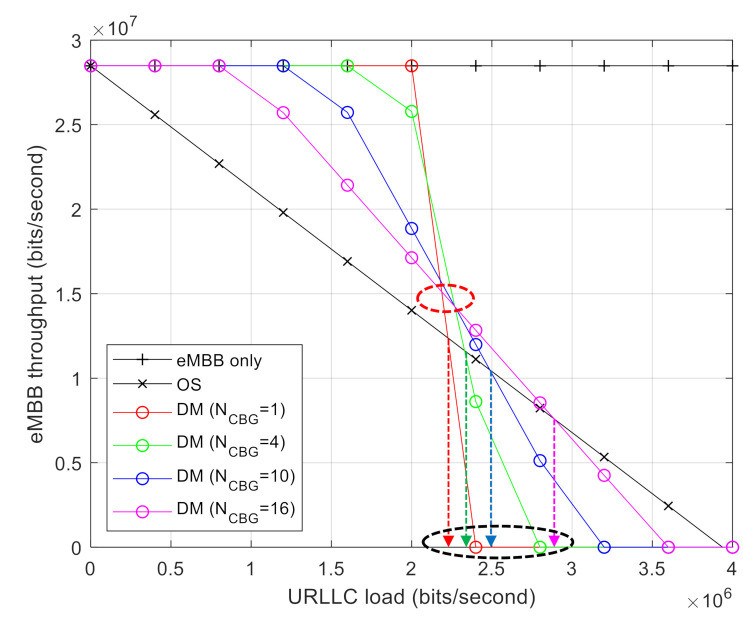
eMBB throughput comparison with different numbers of CBG $N_{CBG}$.

Figure 6 shows the capacity regions of DM and OS when the number of CBGs, $N_{CBG}$, for DM is varied. The size of the CBGs decreases with increasing $N_{CBG}$ for a given TB size. For a smaller CBG size, the amount of puncturing URLLC traffic over the CBG is reduced; however, the error-correcting capability of CBG *l* is also reduced. As the number of CBGs increases, it is possible that one of them is successfully transmitted even if the error-correcting capability of each CBG is reduced. As indicated by the red circles in Figure 6, the performance is degraded when $N_{CBG}$ is large, i.e., *l* is reduced under a low URLLC load. The opposite performance is observed under a high URLLC load, as a large $N_{CBG}$ results in a diversity gain. Furthermore, $D_{threshold}$ increases with an increase in $N_{CBG}$. This implies that a greater number of CBGs leads to a higher DM gain under a high URLLC load.

## 5. Conclusions

In this paper, we propose a dynamic BWP allocation scheme that switches between two multiplexing methods, dynamic multiplexing (DM) and orthogonal slicing (OS). It aims at minimizing an impact of URLLC traffic on eMBB traffic for a UAV network in which diverse QoS requirements are imposed to receive highly reliable autonomous flight messages with low latency for command and control while receiving video streaming services over downlink broadband traffic. To implement efficient BWP allocation, this paper presents a comprehensive performance analysis to identify capacity region by considering the effect of physical layer parameters, such as modulation and coding scheme (MCS) level and code block group size on DM and OS. It was shown that OS is effective for improving the eMBB throughput subject to the URLLC latency constraint when URLLC traffic is deterministic and predictable, as opposed to DM, which has a limited error-correcting capability against the puncturing effect of URLLC. Furthermore, the relative MCS level of eMBB and URLLC is a critical factor in determining the durability of eMBB traffic against the puncturing effect. In fact, it has been quantified by a switching threshold of the URLLC load expressed in an approximate closed form, which can serve as a reference for dynamic BWP allocation. As future work, it would be interesting to generalize a proposed scheme considering implementation issues, such as BWP switching delay and the effect of model inaccuracy.

## Figures and Tables

**Table 1:** Use Cases and Their Performance Requirements for URLLC.

Service Scenario	Reliability	Latency
AR/VR	$1-10^{-5}$	1 to 4 ms (user-plane)
Factory Autonmation	$1-10^{-6}$	1 ms (user-plane)
Transport Industry	$1-10^{-5}$	3 or 7 ms (user-plane)
Power Distribution	$1-10^{-6}$	3 or 6 ms (user-plane)
Command and Control of UAV Network	$1-10^{-3}$	10, 40, or 140 ms (end-to-end)

**Table 2:** MCS index table for Physical Downlink Shared Channel [22].

MCS Index $I_{\mathrm{MCS}}$	Modulation Scheme	Code Rate ×[1024]	Spectral Efficiency
0	QPSK	120	0.2344
1	QPSK	157	0.3066
2	QPSK	193	0.3770
3	QPSK	251	0.4902
4	QPSK	308	0.6016
5	QPSK	379	0.7402
6	QPSK	449	0.8770
7	QPSK	526	1.0273
8	QPSK	602	1.1758
9	QPSK	679	1.3262
10	QPSK	340	1.3281
11	16QAM	378	1.4766
12	16QAM	434	1.6953
13	16QAM	490	1.9141
14	16QAM	553	1.1602
15	16QAM	616	2.2403
16	16QAM	658	2.5703
17	64QAM	438	2.5664
18	64QAM	466	2.7305
19	64QAM	517	3.0293
20	64QAM	567	3.3223
21	64QAM	616	3.6094
22	64QAM	666	3.9023
23	64QAM	719	4.2129
24	64QAM	772	4.5234
25	64QAM	822	4.8164
26	64QAM	873	5.1152
27	64QAM	910	5.3320
28	64QAM	948	5.5547

## Appendix A. Proof of Theorem 1

Let $D_{threshold}$ denote the threshold value of the URLLC load *D*, at which equality holds in (9). Meanwhile, $D_{threshold}$ always exists when $\delta_{CBG}$ has a value between l−ε and l+ε. When $\delta_{CBG}$ is smaller than l−ε, the throughput of an eMBB CBG is the same as that without puncturing. In contrast, when $\delta_{CBG}$ exceeds l+ε, the throughput of an eMBB CBG is zero. Therefore, the inequality in (9) can be re-written as

(A1) $$\frac{1}{2}+\frac{1}{2\varepsilon }\left(l-\delta_{CBG}\right)\leq 1-\frac{D}{b(I_{MCS}^{URLLC})}\cdot \frac{1}{\Phi }$$

By substituting (3) into (A1) and rearranging it for *D*,

(A2) $$D\leq \left(\varepsilon -l\right)\left(\frac{N_{CBG}\cdot \Phi \cdot b\left(I_{MCS}^{URLLC}\right)}{2\varepsilon N_{CBG}-\phi_{u}\cdot b\left(I_{MCS}^{\left(u\right)}\right)}\right)$$

As ε approaches zero, the right-side term of (A2) is given as -4.6cm0cm

(A3) $$\lim_{\varepsilon \to 0}\;\left(\varepsilon -l\right)\left(\frac{N_{CBG}\cdot \Phi \cdot b\left(I_{MCS}^{URLLC}\right)}{2\varepsilon N_{CBG}-\phi_{u}\cdot b\left(I_{MCS}^{\left(u\right)}\right)}\right)=\frac{l\Phi b\left(I_{MCS}^{URLLC}\right)N_{CBG}}{\phi_{u}b\left(I_{MCS}^{\left(u\right)}\right)}=N_{CBG}\cdot l\cdot \left(\frac{\Phi }{\phi_{u}}\right)\cdot \left(\frac{b(I_{MCS}^{URLLC})}{b(I_{MCS}^{\left(u\right)})}\right)$$

Consequently, the switching threshold, $D_{threshold}$, for URLLC traffic load is given by

(A4) $$D_{threshold}=N_{CBG}\cdot l\cdot \left(\frac{\Phi }{\phi_{u}}\right)\cdot \left(\frac{b(I_{MCS}^{URLLC})}{b(I_{MCS}^{\left(u\right)})}\right)$$
